# A Superior Cerebellar Convexity Two-Part Craniotomy to Access the Paramedian Supra and Infratentorial Space: Technical Note

**DOI:** 10.7759/cureus.664

**Published:** 2016-06-30

**Authors:** Tene Cage, Arnau Benet, John Golfinos, Michael W. McDermott

**Affiliations:** 1 Department of Neurological Surgery, University of California, San Francisco; 2 Department of Otolaryngology, University of California, San Francisco; 3 Department of Neurosurgery, NYU Langone Medical Center

**Keywords:** posterior fossa, cerebellar convexity, transverse sinus, asterion

## Abstract

A craniotomy over the superior cerebellar convexity for approaches to this region typically involves a small infratentorial craniotomy and then drilling down of the bone to expose some portion of the transverse/sigmoid sinuses. The authors describe the anatomy of the region and the method for a two-part paramedian occipital and suboccipital craniotomy (supra and infratentorial) that may have time-saving, safety, and cosmetic advantages. For this technique, a supratentorial craniotomy is used to expose the transverse sinus from above, and subsequently, dissection across the sinus over the cerebellar convexity can be done under direct vision. The two bone pieces are joined on the inner table side while plates for fixation above the superior nuchal line can be counter-sunk to avoid post-operative pain from the prominence of screws. There is no need for cranioplasty materials since there is no burring down of bone for adequate exposure of the transverse sinus. The technique has been used by two senior surgeons over the years convincing them of the speed, safety, and utility of the technique. Here, the authors present a single example of the technique.

## Introduction

The combined paramedian superior cerebellar convexity, transverse sinus, and cerebellar regions are critical areas to safely access in neurosurgery. The neurosurgeon must routinely use this approach over the cerebellum to the tentorial incisura and brainstem region for tumors straddling the tentorium, for tumors attached to the cerebellar surface of the tentorium adjacent to the transverse sinus, for intra-axial pathology in the cerebellar hemisphere, for vascular malformations in the region, and for arachnoid cysts [1-3]. The traditional approach to accessing this area involves several steps. First, the occipital bone over the superior cerebellar convexity is exposed and an infratentorial posterior fossa craniotomy is carried out. For safety considerations, the burr holes to start the infratentorial craniotomy are placed several millimeters away from the inferior edge of the transverse sinus, and once the infratentorial craniotomy bone is removed, the remaining occipital bone covering the sinus is drilled using cutting or diamond burrs and rongeurs. Next, the inferior to the entire portion of the transverse sinus is unroofed from below by drilling off the bone superior to the craniotomy. Drilling the bone to expose the sinus requires significant time and carries risk of sinus laceration.

We describe an alternative technique to expose the superior cerebellar convexity, transverse sinus, and posterior fossa using a two-part craniotomy. This technique obviates the need for laborious drilling over the transverse sinus. The two-part craniotomy offers a more expeditious, safer, and more cosmetically favorable approach to the supracerebellar corridor when compared to the traditional combined craniotomy and craniectomy to expose the supra and infratentorial convexities. We present a patient with a complex and symptomatic supracerebellar arachnoid cyst that was successfully fenestrated using this modified craniotomy to illustrate the technique and results.

## Technical report

### Case presentation

**Figure 1:**
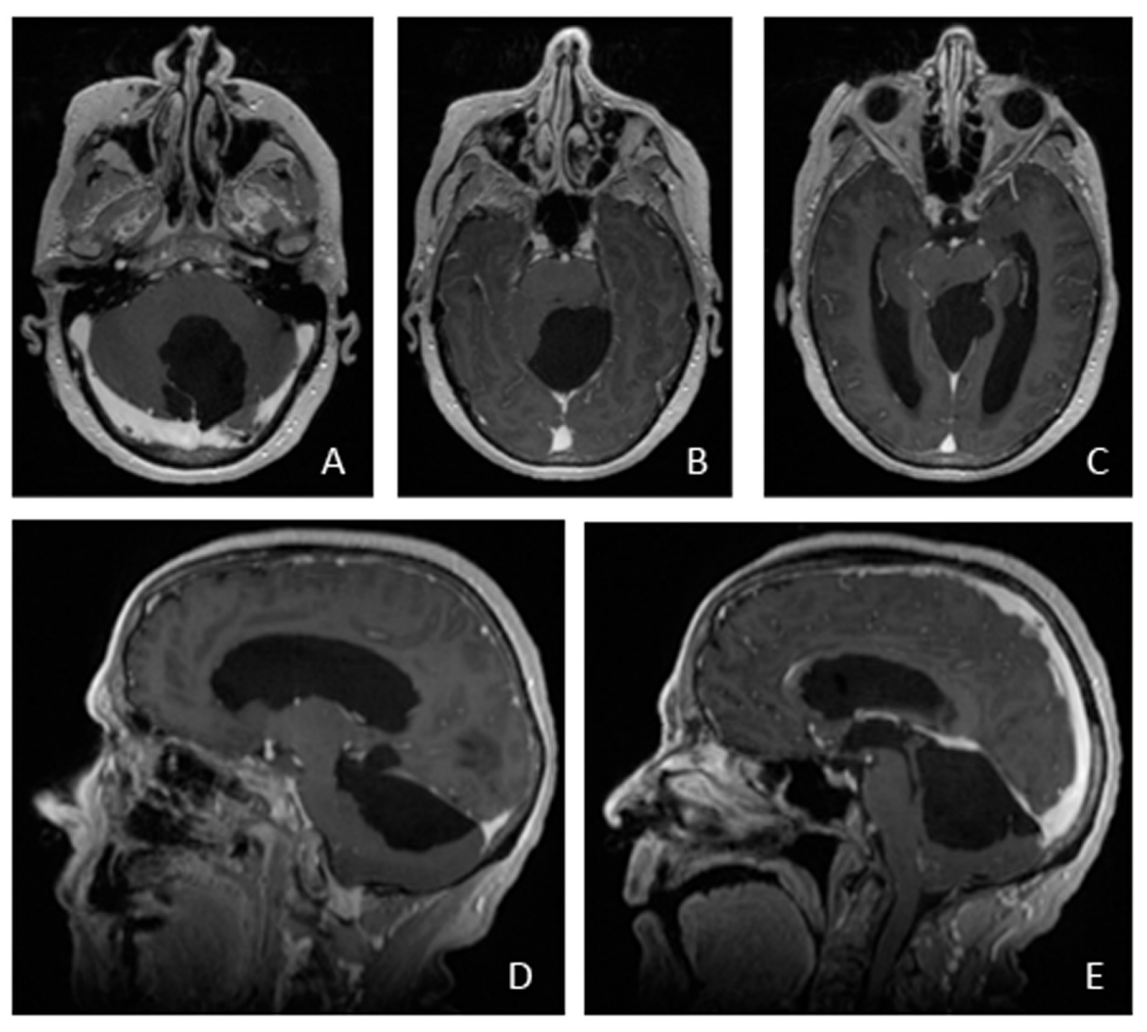
Preoperative MRI Scans Axial (A-C) and sagittal (D-E) T1-weighted brain MRI with gadolinium contrast images illustrating a large 6.4 x 4.3 x 3.8 cm multilobulated arachnoid cyst in the supracerebellar cistern and quadrigeminal cistern.

A 73-year-old woman presented with nausea, increasing confusion, ataxia, and mechanical falls. MRI imaging revealed a 6.4 x 4.3 x 3.8 cm multilobulated fluid collection in the supracerebellar cistern and quadrigeminal cistern consistent with a complex arachnoid cyst (Figure 1). The patient agreed to participate and was explained the nature and objectives of this study, and informed consent was formally obtained. No reference to the patient's identity was made at any stage during data analysis or in the report. 

**Figure 2:**
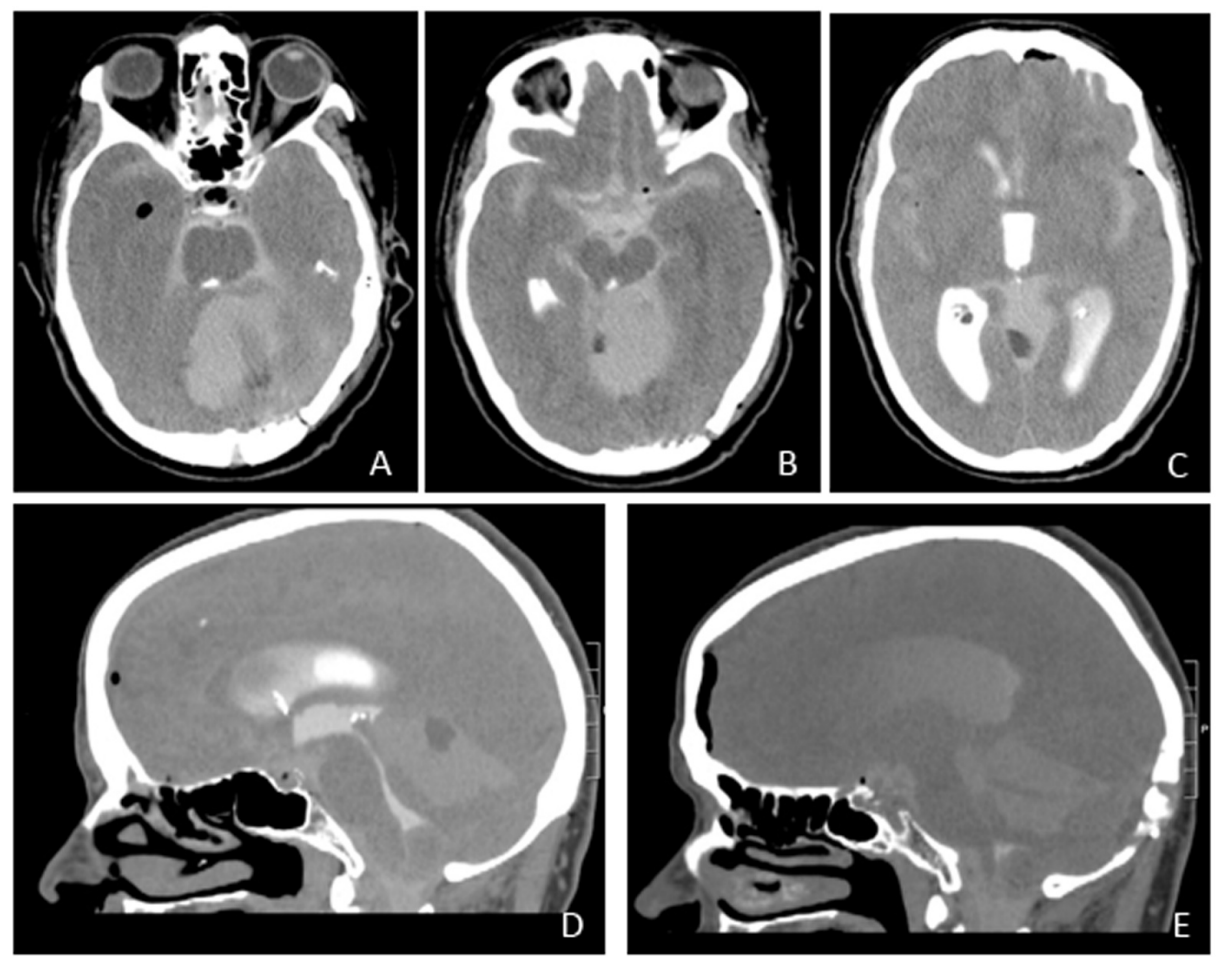
Postoperative CT Cisternogram Images Axial (A-C) and sagittal (D-E) CT cisternogram after surgical fenestration of an arachnoid cyst indicates communication between the CSF of the ventricles and the arachnoid cyst.

Because she had symptomatic hydrocephalus due to the mass effect of the arachnoid cyst, an external ventricular drain was placed into the right lateral ventricle at the time of admission. She was then taken for definitive surgical treatment with decompression and fenestration of the cyst through a superior cerebellar approach as described in detail below. She tolerated the procedure well and post-operatively recovered in the intensive care unit. Her symptoms resolved, and after a cisternogram confirmed that the cerebral aqueduct was patent with good fenestration of the arachnoid cyst (Figure 2), the external ventricular drain was removed on postoperative day two.

The patient did well, symptoms resolved, and on postoperative day eight she was discharged in good condition.

### Operative anatomical considerations: anatomy of the squamous part of the occipital bone

The occipital bone is divided into basilar, condylar, and squamous parts. Although all three parts are relevant to neurosurgery, only the squamous part of the occipital bone (SQOB) is involved in the approaches to the cerebellar convexity and the transverse sinus. The surgical anatomy of the SQOB is therefore described to provide the rationale for this surgical approach. The shape of the SQOB resembles that of a simple pentagon. The superior vertex of the SQOB is called the lambda, which corresponds to the confluence of the paired lambdoid sutures (between the parietal and occipital bones) and the sagittal suture (between the parietal bones). The lambdoid sutures descend laterally to meet the mastoid part of the temporal bone. At its most lateral extension (the lateral angles of the pentagon), the SQOB meets both the parietal bone (lambdoid suture) and the temporal bone through the parietomastoid suture. The intersection of the lambdoid and occipitomastoid sutures is called the asterion. The asterion may be used surgically to infer the inferior aspect of the transition from transverse to sigmoid sinus. In addition, a projected *imaginary* line between both asterion landmarks corresponds to the inferior border of the transverse sinus and, indirectly, the level of the tentorium. Below the asterion, the occipitomastoid suture descends until the SQOB transitions into the condylar part, at the condylar fossa, which contains the condylar canal. The base of the imaginary pentagon contains the posterior half of the foramen magnum, the posterior vertex of which is called opisthion. The exocranial surface of the SQOB is densely shaped by the insertion lines of the nuchal and suboccipital muscles. The external occipital protuberance (also known as inion) is a palpable protrusion in the middle of the SQOB, and serves as a landmark for the confluence of sinuses on the endocranial surface (approximately 1 cm above the inion). Two horizontal lines project laterally from the inion, the highest (also known as supreme) nuchal line superiorly and the superior nuchal line inferiorly. The transverse sinus is located between both lines within the endocranial surface. Superior to the inion is the opistocranion, the highest point of the occipital vault in the exocranial surface of the SQOB. Inferior to the inion, the inferior nuchal line crosses the SQOB horizontally, below the superior nuchal line, and it is shaped by the insertion of the suboccipital muscles below it. A single vertical ridge, the external occipital crest, emerges from foramen magnum and may be used to infer the endocranial location of the occipital sinus and falx cerebelli as well as the midline.

The endocranial surface of the SQOB is greatly shaped by the venous sinuses. When viewed from the inside, the SQOB contains a carved cross and four convex depressions. The internal occipital protuberance is a prominence at the center of the SQOB, emerging inferior to the confluence of sinuses. The confluence of sinuses, or torcula of Herophilos, is the anatomical space where the transverse, superior sagittal, occipital, and straight sinuses meet. In the axial plane, the tentorium forms the transverse sinuses posteriorly, the superior petrosal sinuses laterally, and divides the intracranial volume into supra and infratentorial spaces. In the sagittal plane, the cerebral hemispheres are divided by the cerebral falx supratentorially, and the falx cerebelli infratentorially. Of importance for the surgical technique described in this report is that the transverse sinuses carve a sulcus in the endocranial surface of the SQOB. These sulci are bounded by a ridge of bone, which severely interferes with the footplate of the drill when a 'one piece' supra and infratentorial craniotomy is attempted, as the footplate is trapped on this bony ridge. Forceful maneuvering of the drill at this region risks sinus damage. A two-piece craniotomy, which opens a bone window superior to the transverse sinus first, allows for dissection of the sinus from its sulcus under direct view and guarantees preservation of the sinus. Next, an inferior craniotomy is turned with the sinus retracted and under direct view, allowing for a safe and efficient craniotomy overall. The bony ridge and convexity of the posterior fossa are more easily crossed after dural sinus dissection, as described above, in the cranial to caudal direction thus arguing to perform the supratentorial craniotomy rather than the infratentorial craniotomy first.

### Operative technique

**Figure 3:**
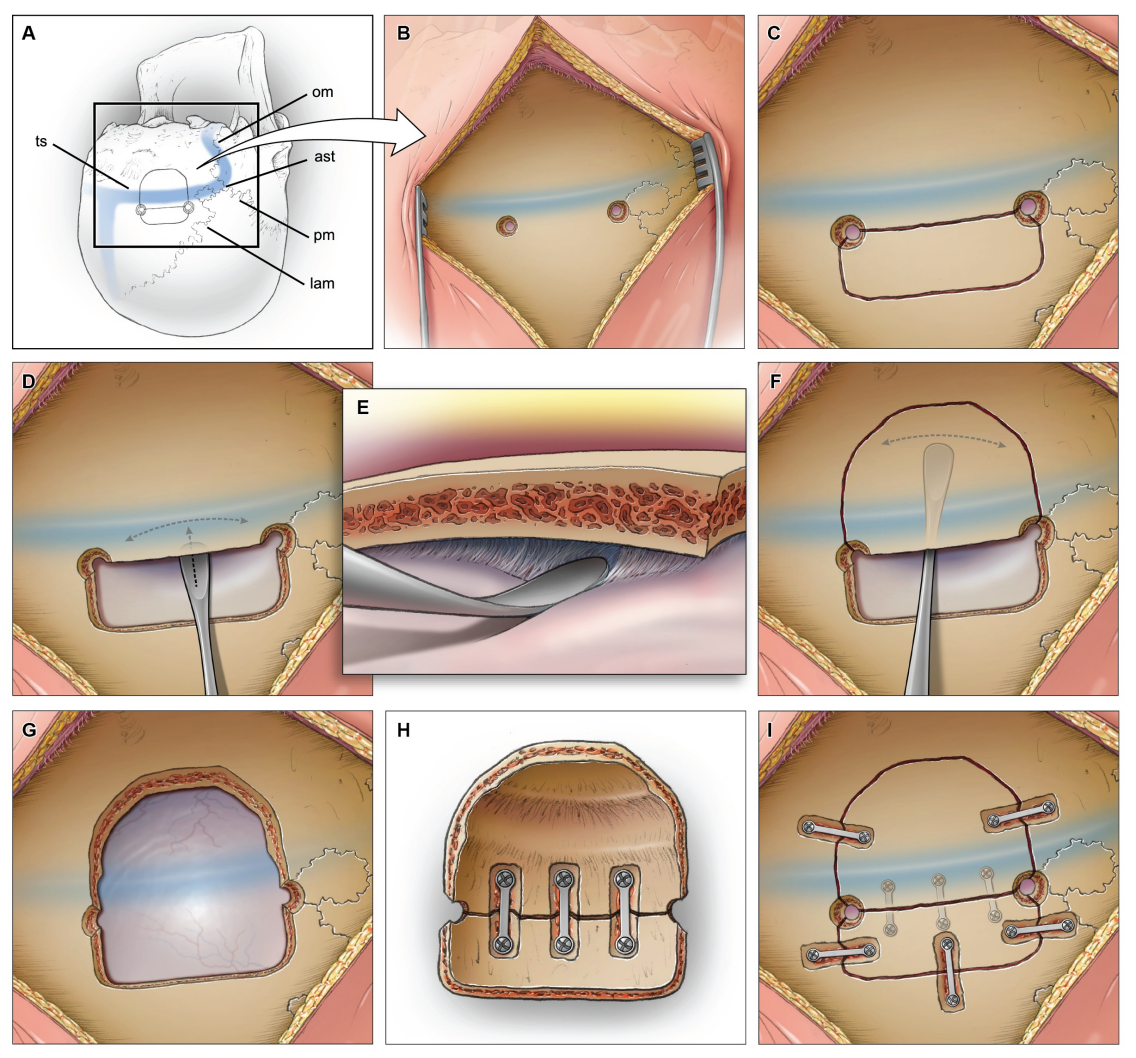
Operative Technique Schematic panel representation of the operative steps used to perform the two-part supracerebellar craniotomy to access the supra and infratentorial space. om: occipitomastoid suture ast: asterion suture pm: parietomastoid suture lam: lambdoid suture ts: transverse sinus

**Figure 4:**
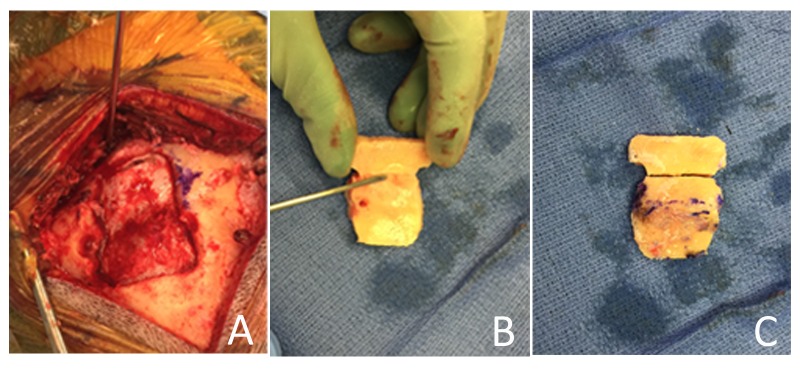
Intraoperative Images of Craniotomy (A) The two-part craniotomy is elevated to reveal intact dura spanning the transverse sinus. (B) The inner concavity of the two part bone flap is seen. The Penfield #4 instrument points to the imprint of the transverse sinus. (C) The two bone pieces have been fixed to each other on the internal surface and here, the external convexity is restored with an excellent anatomic cosmetic result.

A linear incision is used to expose the region of the occipital bone above and below the transverse sinus. Image guidance is used to define the margin of the sinus so that two burr holes can be accurately placed in the supratentorial space 3-5 mm above the sinus (Figure 3A-3B). The dura is dissected using a right-angled Penfield #4, and a small semilunar bone flap is elevated (Figure 3C). Then under direct vision the dura is dissected from the bone, crossing the bony gutter housing the sinus, onto the posterior fossa convexity dura (Figure 3D-3E). The dissection of posterior fossa dura can be easily extended medially to the midline occipital crest, laterally to the sigmoid sinus, and inferiorly toward the foramen magnum. The footplate attachment of the drill is then used to elevate the secondary bone flap (Figure 3F-3G, Figure 4A). The concavity in the bone can be seen on the inner surface (Figure 4B), and to prepare the bone for surgical closure, the two pieces are connected one to another on the inner table side using plates and screws (Figure 3H). Above the nuchal line plates and screws are recessed in the outer table so as to prevent a palpable prominence which may cause post-operative discomfort (Figure 3I, Figure 4C). No additional cranioplasty is required.

The senior authors here (MWMcD, JG) have used the technique described herein for posterior fossa convexity craniotomies over the past 23 years and believe it is easier, safer, and faster than traditional approaches.

## Discussion

The superior cerebellar craniotomy is an important surgical technique for the cranial neurosurgeon to have in her or his arsenal of posterior fossa approaches. This approach is critical to access and treat a variety of intracranial pathologies including pineal region tumors, inferiorly projecting tentorial meningiomas, vascular lesions such as arteriovenous malformations, and arachnoid cysts of the posterior fossa as described in this case [2, 4-5]. Each of these pathologies must be accessed via a surgical corridor that navigates across the transverse sinus superiorly and often requires a deep and narrow trajectory along the superior surface of the cerebellum. In order to safely gain the necessary exposure to this region, the transverse sinus must be visualized and uncovered. However, removal of bone in this region puts the transverse sinus at risk and therefore exposes the patient to a risk of vigorous intraoperative blood loss. In addition, the cerebellar surface is also at risk of injury during bone removal in this region.

To mitigate injury to the venous sinus and to the parenchyma, we have modified the standard infratentorial craniotomy with associated craniectomy over the transverse sinus technique to gain access to the supracerebellar infratentorial space. The standard cranial access technique of an infratentorial craniotomy with associated craniectomy to visualize the transverse sinus carries a true risk of sinus laceration during direct drilling over the transverse sinus during the craniectomy portion. Instead, we offer a two-part craniotomy technique spanning the transverse sinus safely. The first bone flap above the transverse sinus can be made safely by dissecting the dura away from the bone without concern for venous injury. Then, with the first bone piece removed, the transverse sinus is dissected free from bone under direct visualization, and therefore, can be protected from injury. Once the surgeon is confident about the bone and sinus separation, the second bone flap across the sinus can then be turned safely while visualizing the cranial drill foot plate under the bone with the sinus free below. Jia et al., have described a similar two-part craniotomy across the lateral transverse sinus and sigmoid sinus junction with excellent outcomes to protect the sinus from injury [6]. This technique is also used routinely to protect the superior sagittal sinus when a bifrontal, biparietal, or bioccipital craniotomy is required. Crossing the major venous sinuses in this fashion decreases the potential for injury to the sinus as the bone is removed. And in addition, if the sinus is injured during flap elevation, venous bleeding can be more easily controlled with the sinus exposed for repair.

Cosmetically, our modified cranial opening allows for the patient’s autologous bone to be replaced entirely. This allows for a good cosmetic outcome and mitigates the need for cranioplasty materials that are required to repair the bone defect and protect the exposed transverse sinus that results from the standard craniotomy and craniectomy technique. Such cranioplasty materials including titanium mesh and methacrylate may carry a complication risk including infection or visible cosmetic defect that vary in the literature from 0-62% [7]. However, our technique does require a larger skin incision to expose both the supratentorial and infratentorial spaces. Additionally, because the supratentorial bone is removed, there is the potential for contusion or injury to the occipital lobe during the exposure which is not true in a traditional approach.

Our two-piece craniotomy technique offers an alternative to the traditional infratentorial craniotomy with craniectomy over the sinus while providing a safer and more controlled approach to the superior cerebellar cranial window. The two senior authors have used this technique many times with good outcomes and are able to successfully protect the integrity of the transverse sinus, create the appropriate surgical corridor to access lesions in supracerebellar infratentorial space, and to reconstruct the cranial defect without foreign cranioplasty materials following surgery.

## Conclusions

The authors present the superior cerebellar convexity two-part craniotomy as an alternative to the traditional infratentorial craniotomy and craniectomy for surgical approaches to this region. Direct blunt dissection and visualization of the transverse sinus from above improves the safety of sinus exposure. The ability to remove the infratentorial bone without craniectomy may improve the speed of an approach to this region, and the repair/closure of the craniotomy does not require additional materials.
